# Anemia and red blood cell transfusion practice in prolonged mechanically ventilated patients admitted to a specialized weaning center: an observational study

**DOI:** 10.1186/s12890-019-1009-1

**Published:** 2019-12-18

**Authors:** Alessandro Ghiani, Alexandros Sainis, Georgios Sainis, Claus Neurohr

**Affiliations:** 1 Department of Pneumology and Respiratory Medicine, Schillerhoehe Lung Clinic (Robert Bosch Hospital GmbH, Stuttgart), Solitudestr. 18, 70839 Gerlingen, Germany; 2 Athens, Greece; 3 Cologne, Germany; 4 Munich, Germany

**Keywords:** Anemia, Transfusion, Mechanical ventilation, Weaning, Mortality, Nosocomial infections

## Abstract

**Background:**

The impact of anemia and red blood cell (RBC) transfusion on weaning from mechanical ventilation is not known. In theory, transfusions could facilitate liberation from the ventilator by improving oxygen transport capacity. In contrast, retrospective studies of critically ill patients showed a positive correlation of transfusions with prolonged mechanical ventilation, increased mortality rates, and increased risk of nosocomial infections, which in turn could adversely affect weaning outcome.

**Methods:**

Retrospective, observational study on prolonged mechanically ventilated, tracheotomized patients (*n* = 378), admitted to a national weaning center over a 5 year period. Medical records were reviewed to obtain data on patients’ demographics, comorbidities, blood counts, transfusions, weaning outcome, and nosocomial infections, defined according to the criteria of the U.S. Centers for Disease Control and Prevention. The impact of RBC transfusion on outcome measures was assessed using regression models.

**Results:**

Ninety-eight percent of all patients showed anemia on admission to the weaning center. Transfused and non-transfused patients differed significantly regarding disease severity and comorbidities. In multivariate analyses, RBC transfusion, but not mean hemoglobin concentration in the course of weaning, was independently correlated with weaning duration (adjusted β 12.386, 95% CI 9.335–15.436; *p* <  0.001) and hospital length of stay (adjusted β 16.116, 95% CI 8.925–23.306; *p* <  0.001); there was also a trend toward increased hospital mortality (adjusted odds ratio [OR] 2.050, 95% CI 0.995–4.224; *p* = 0.052), but there was no independent correlation with weaning outcome or nosocomial infections. In contrast, hemoglobin level on the day of admission to the weaning center was independently associated with hospital mortality (adjusted OR 0.956, 95% CI 0.924–0.989; *p* = 0.010), appearing significantly elevated at values below 8.5 g/dl (AUC 0.670, 95% CI 0.593–0.747; *p* <  0.001).

**Conclusions:**

A high percentage of prolonged mechanically ventilated patients showed anemia on admission to the weaning center. RBC transfusion was independently correlated with worse outcomes. Since transfused patients differed significantly regarding their clinical characteristics and comorbidities, RBC transfusion might be an indicator of disease severity rather than directly impacting patient prognosis.

## Background

Intubation and mechanical ventilation is a life-saving procedure for patients presenting with severe acute respiratory failure. The majority of patients can be weaned promptly on recovery and resolution of the underlying condition. However, 7–14% of patients remain ventilated longer than 7 days [1, 2], which is frequently followed by tracheostomy and transferal to specialized weaning and rehabilitation centers, with variable outcomes reported [3, 4]. In virtually all critically ill patients hemoglobin levels decline below normal limits during their stay in the intensive care unit (ICU). Therefore, anemia is a very common comorbid condition at the time of admission to the weaning center [5]. In patients receiving long-term mechanical ventilation, the presence of anemia may interfere with their ability to wean from ventilatory support [6]. Nevertheless, although it seems reasonable to speculate that improving oxygen delivery, to overcome the increased workload on respiratory muscles during spontaneous breathing trials (SBT), will consequently facilitate weaning from mechanical ventilation, the hemoglobin threshold for transfusion that can best help achieve this goal is still undetermined. Only a few small case studies have investigated this question [7, 8], with no clear recommendations made in the existing guidelines [1, 9]. In the 1980s and later, studies were published that were indicative of an immunomodulatory effect [10] of red blood cell (RBC) transfusions in critically ill patients, describing an association with nosocomial infections (NI) [11–16], which in turn could adversely affect patients’ outcome.

The aim of the present study was to describe the incidence and severity of anemia and to investigate the impact of RBC transfusion on weaning outcome, hospital mortality, and nosocomial infection rates in prolonged mechanically ventilated, tracheotomized patients, admitted to a specialized weaning center.

## Methods

This is a retrospective, single-center, observational cohort study at a national weaning center in Germany. The 12-bed ward was established in 2006 and is part of the specialized lung clinic at Schillerhoehe (Gerlingen). The weaning unit is equipped to provide invasive (by tracheostomy tube) and non-invasive ventilatory support. Since the establishment of the unit, it has taken referrals from intensive care units across Germany. Main preconditions for referral of a patient were hemodynamic stability without need for vasopressors or inotropic agents, wakefulness without need for continuous sedation, and invasive mechanical ventilation by tracheostomy tube with a positive end-expiratory pressure less than 10 cmH_2_O and a fraction of inspired oxygen less than 0.6.

The study was approved by the local ethics committee, the need for informed consent was waived (ethics committee of the State Chamber of Physicians of Baden-Wuerttemberg).

### Patient selection

All consecutive patients transferred to the weaning center between December 2011 and December 2016 were identified. Patients were included if they were referred because of evident failure to wean from mechanical ventilation and met the criteria of prolonged weaning, classified as Category 3 as defined by Boles and colleagues, based on the overall duration of weaning as well as the number of spontaneous breathing trials required to liberate the patient from the ventilator [1]. Patients were excluded if a tracheostomy existed before the acute illness or if a transfer to another hospital had to take place in the course of weaning and weaning outcome thus remained uncertain.

### Ventilator weaning

Weaning was systematically performed according to the recommendations from the statement by Boles and colleagues [1] as well as to the national guidelines on prolonged weaning [9]. The process included protocol-based increasing periods of unassisted breathing through a tracheostomy collar, usually starting with a 30 min SBT, and then advancing with at most 2 h per day. Between T-piece ventilation, patients were mechanically ventilated in the assisted-controlled ventilator mode in order to recover from the imposed work of breathing during their SBT [17]. Extensive use of non-invasive ventilation (NIV), avoidance of sedatives, attention to nutrition, proactive physiotherapy, and optimal therapy of comorbidities constituted the approach to weaning. RBC transfusion in the course of weaning was regulated in an in-house protocol, based on national recommendations on the use of blood products [18]. In principle, a restrictive transfusion strategy was recommended with a transfusion trigger of hemoglobin less than 7.0 g/dl, whereas it was 8.0 g/dl in patients with cardiac comorbidities (coronary artery disease, cardiac surgery patients).

At the end of the weaning process, patients were further subclassified into Categories 3a, 3b, or 3c, based on the German guideline on prolonged weaning [9]. Category 3a describes persistent spontaneous breathing for more than 72 h without concomitant clinical and laboratory signs of chronic ventilatory insufficiency. Category 3b represents the transition from invasive ventilation to non-invasive home ventilation. Category 3c is defined as weaning failure, corresponding to transition to permanent home ventilation by tracheostomy tube or death on ventilation in the course of weaning.

### Data collection

Data were retrospectively collected from referral letters and hospitals’ electronic medical record and chart system. Patients’ baseline characteristics on admission, such as demographic data and comorbidities, all hemoglobin values of each patient in the course of weaning as well as the number of transfused RBCs and transfusion episodes with the corresponding pre-transfusion hemoglobin values were collected. Anemia was defined as hemoglobin concentration < 14.0 g/dl in males and < 12.0 g/dl in females, respectively [19]. Patients’ records were reviewed for new nosocomial infections more than 2 days after admission to the weaning unit and up to 48 h after completion of weaning, defined according to the revised 2008 criteria of the U.S. Centers for Disease Control and Prevention (CDC) [20]. Further data included weaning duration, hospital length of stay, and hospital mortality rates.

Additional details on data collection can be found in the online supplement [see Additional file 1].

### Statistical analysis

Descriptive and frequency statistics were used to summarize patients’ demographics and baseline characteristics. Differences in categorical variables between groups were analyzed using the Chi-square test or Fisher’s exact test, as appropriate. The Student’s *t*-test was used to examine differences in continuous variables.

Pearson correlation coefficient was used to analyze a possible association between the number of RBC transfusions and the number of nosocomial infections.

Univariate and multivariate regression analyses were used to assess the impact of RBC transfusion, mean hemoglobin concentration in the course of weaning, hemoglobin concentration on the day of admission to the weaning center, and further clinical parameters describing disease severity (APACHE-II score, Charlson comorbidity index, weaning duration), on weaning outcome measures, hospital mortality, and infectious complications. Results were adjusted for patients’ baseline clinical characteristics, transfusion of FFP/platelets, and bleeding complications in the course of weaning. The results for nosocomial infections were adjusted for weaning duration.

The predictive performance of the hemoglobin concentration on the day of admission regarding survival was assessed with the receiver operating characteristic (ROC) curve, and the area under the ROC curve (AUC), which summarizes the performance in predicting hospital mortality, was calculated.

We considered *p* <  0.05 to be statistically significant for all tests performed.

## Results

In the described period, 378 patients met the inclusion criteria; a further 14 patients were excluded. The total population was divided into transfused (T) and non-transfused (NT) patients; there were 168 (44.4%) patients in the T group and 210 (55.6%) patients in the NT group. Table 1 shows the baseline clinical characteristics of all patients and between the two groups on admission to the weaning center.

**Table 1:** Clinical characteristics on admission to the weaning center

	All patients (*n* = 378)	Transfused (*n* = 168)	Non-transfused (*n* = 210)	*P* value^*d*^
Clinical characteristics				
Age (years)	69.3 (± 12.0)	70.6 (± 10.2)	68.3 (± 13.2)	0.058^*a*^
Gender (male)	229 (60.6)	100 (59.5)	129 (61.4)	n.s.^*b*^
Body mass index (kg/m^2^)	27.1 (± 7.7)	25.7 (± 6.7)	28.2 (± 8.3)	0.001^*a*^
Smoking history	165 (43.7)	68 (40.5)	97 (46.2)	n.s.^*b*^
APACHE-II	16.2 (± 5.0)	17.1 (± 5.0)	15.5 (± 5.0)	0.002^*a*^
Albumin (g/dl)	2.0 (± 0.5)	1.9 (± 0.5)	2.1 (± 0.5)	0.002^*a*^
No. of patients with anemia	372 (98.4)	167 (99.4)	205 (97.6)	n.s.^*b*^
Hemoglobin on the day of admission (g/dl)	9.0 (± 1.3)	8.5 (± 1.0)	9.4 (± 1.3)	< 0.001^*a*^
Ventilator days on admission	28.4 (± 19.9)	31.4 (± 22.0)	26.0 (± 17.7)	0.010^*a*^
Time from ETI to tracheostomy (days)	10.2 (± 7.0)	11.0 (± 7.7)	9.5 (± 6.4)	0.035^*a*^
ECLA	20 (5.3)	10 (6.0)	10 (4.8)	n.s.^*b*^
Comorbidities				
No. of comorbidities per patient	2.1 (± 1.6)	2.1 (± 1.5)	2.0 (± 1.6)	n.s.^*a*^
Charlson comorbidity index	6.2 (± 2.6)	6.7 (± 2.2)	5.9 (± 2.8)	0.001^*a*^
COPD	103 (27.2)	37 (22.0)	66 (31.4)	0.041^*b*^
Coronary artery disease	113 (29.9)	59 (35.1)	54 (25.7)	0.047^*b*^
Left ventricular dysfunction (systolic)	83 (22.0)	46 (27.4)	37 (17.6)	0.023^*b*^
Renal insufficiency (GFR < 60 ml/min)	88 (23.3)	52 (31.0)	36 (17.1)	0.001^*b*^
Hepatopathy	22 (5.8)	12 (7.1)	10 (4.8)	n.s.^*b*^
Diabetes mellitus	114 (30.2)	45 (26.8)	69 (32.9)	n.s.^*b*^
Neuromuscular disease	21 (5.6)	6 (3.6)	15 (7.1)	n.s.^*b*^
Interstitial lung disease	21 (5.6)	8 (4.8)	13 (6.2)	n.s.^*b*^
Malignancy (active)	39 (10.3)	20 (11.9)	19 (9.1)	n.s.^*b*^
Immunosuppression	49 (13.0)	20 (11.9)	29 (13.8)	n.s.^*b*^
Cause of acute respiratory failure				
Pneumonia	124 (32.8)	49 (29.2)	75 (35.7)	n.s.^*b*^
Sepsis (incl. Septic shock)	33 (8.7)	16 (9.5)	17 (8.1)	n.s.^*b*^
Acute exacerbation of COPD	36 (9.5)	6 (3.6)	30 (14.3)	< 0.001^*b*^
Cardiac failure	12 (3.2)	2 (1.2)	10 (4.8)	0.049^*b*^
Cardiopulmonary resuscitation	31 (8.2)	20 (11.9)	11 (5.2)	0.019^*b*^
Surgery	94 (24.9)	57 (33.9)	37 (17.6)	< 0.001^*b*^
*Cardiac surgery*	39 (10.3)	27 (16.1)	12 (5.7)	0.001^*b*^
*Thoracic surgery*	32 (8.5)	19 (11.3)	13 (6.2)	n.s.^*b*^
*Abdominal surgery*	14 (3.7)	7 (4.2)	7 (3.3)	n.s.^*b*^
*Other*	9 (2.4)	4 (2.4)	5 (2.4)	n.s.^*b*^
Trauma	9 (2.4)	4 (2.4)	5 (2.4)	n.s.^*b*^
Other	39 (10.3)	14 (8.3)	25 (11.9)	n.s.^*b*^

Legend

Continuous variables are presented as mean values (± standard deviation); categorical variables are presented as number (%).

^*a*^: Student’s *t*-test

^*b*^: Chi square test

^*c*^: Fisher’s exact test

^*d*^ P value for differences between transfused and non-transfused patients

*Abbreviations: n.s.* not significant (*p* > 0.05), *APACHE-II* Acute physiology and chronic health evaluation II score, *ETI* Endotracheal intubation, *ECLA* Extracorporeal lung assistance (in acute respiratory failure), *no*. number, *COPD* Chronic obstructive pulmonary disease, *GFR* Glomerular filtration rate

They differed significantly, transfused patients showed higher disease severity with a higher mean APACHE-II score, higher mean Charlson comorbidity index (CCI), and more ventilator days on admission. In the total population, 100% of male patients showed anemia on admission to the weaning center, as did 96% of female patients. Significantly lower hemoglobin levels were found in the T group (8.5 ± 1.0 g/dl and 9.4 ± 1.3 g/dl, respectively; *p* <  0.001).

### RBC transfusion and weaning outcome

Table 2 compares the results between the two groups.

**Table 2:** Red blood cell transfusion and weaning outcome

Result	All patients (*n* = 378)	Transfused (*n* = 168)	Non-transfused (*n* = 210)	*P* value^*e*^
RBC transfusion				
Mean hemoglobin in the course of weaning (g/dl)^*a*^	9.3 (± 1.0)	8.9 (± 0.8)	9.7 (± 1.1)	< 0.001^b^
Pre-transfusion hemoglobin value (g/dl)	7.5 (± 0.5)	7.5 (± 0.5)		–
No. of transfusion episodes	361	361		–
Transfusion episodes per patient	1.0 (± 1.6)	2.2 (± 1.9)		–
No. of transfused RBC units	580	580		–
RBC units per patient	1.5 (± 2.9)	3.5 (± 3.6)		–
Transfusion reactions	0 (0.0)	0 (0.0)		
Transfusion of FFP and/or platelets	8 (2.1)	8 (4.8)	0 (0.0)	< 0.001^c^
Bleeding complications	21 (5.6)	17 (10.1)	4 (1.9)	< 0.001^d^
Gastrointestinal bleeding	12 (3.2)	10 (5.6)	2 (1.0)	n.s.^*d*^
Other bleeding events	9 (2.4)	7 (4.2)	2 (1.0)	n.s.^*d*^
Weaning outcome				
Weaning success (Category 3a/3b)	264 (69.8)	107 (63.7)	157 (74.8)	0.020^*d*^
Category 3a	191 (50.5)	84 (78.5)	107 (68.2)	n.s.^*d*^
Category 3b	73 (19.3)	23 (21.5)	50 (31.8)	0.013^*d*^
Weaning failure (Category 3c)	114 (30.2)	61 (36.3)	53 (25.2)	0.022^*d*^
Invasive HMV	101 (26.7)	51 (30.4)	50 (23.8)	n.s.^*d*^
Death on ventilation	13 (3.4)	10 (5.6)	3 (1.4)	0.016^*d*^
Long-term ventilator dependency^*f*^	174 (46.0)	74 (44.0)	100 (47.6)	n.s.^*d*^
Weaning duration (days)	23.0 (± 16.2)	29.9 (± 20.0)	17.5 (± 9.1)	< 0.001^*b*^
Hospital length of stay (days)	48.0 (± 33.9)	57.7 (± 44.1)	39.0 (± 26.4)	< 0.001^*b*^
Hospital mortality	45 (11.9)	31 (18.5)	14 (6.7)	< 0.001^*d*^

Legend

Results are presented as mean values (± standard deviation), number or number (%).

^*a*^: Mean of all median hemoglobin values of each patient in the course of weaning

^*b*^: Student’s *t*-test

^*c*^: Fisher’s exact test

^*d*^: Chi square test

^*e*^ *P* value for differences between transfused and non-transfused patients

^*f*^ Summary of patients categorized as 3b or 3c–Invasive HMV

*Abbreviations: no.* number, *RBC* packed red blood cells, *n.s*. not significant (*p* > 0.05), *FFP* Fresh frozen plasma, *Invasive HMV* Invasive home mechanical ventilation

In total, 4807 hemoglobin values were recorded in the course of weaning, 3175 in the T group and 1632 in the NT group. The mean of all median hemoglobin values of each patient was significantly lower in the T group than in the NT group (8.9 ± 0.8 g/dl and 9.7 ± 1.1 g/dl, respectively; *p* <  0.001).

In the course of weaning, a total of 361 transfusion episodes were recorded, corresponding to the same number of pre-transfusion hemoglobin values. A total of 580 units of RBCs were transfused, with no transfusion reaction detected in the T group. The mean pre-transfusion hemoglobin concentration was 7.5 ± 0.5 g/dl; 18.8% of pre-transfusion values were lower than 7.0 g/dl, 67.9% were between 7.0 g/dl and 8.0 g/dl, and 11.9% were between 8.0 g/dl and 9.0 g/dl. There was a significant difference in the number of platelets and fresh frozen plasma (FFP) units transfused between the groups, with more products administered in the T group (4.8 and 0.0%, respectively; *p* <  0.001), although the absolute difference was low. In total, 185 patients (48.9%) were transfused during their stay in the ICU. Based on the available data, the number of transfused RBC units per patient before admission did not differ between the two groups (1.27 ± 0.6 and 1.18 ± 0.7, respectively; *p* = 0.156). The same applies to the transfusion of FFP and platelets (0.84 ± 0.9 versus 0.84 ± 0.9; *p* = 0.990).

Patients in the T group had a significant longer duration of weaning, longer hospital length of stay and increased crude hospital mortality. Weaning failure (Category 3c) occurred in a higher percentage of transfused patients, although there was no difference in the proportion of patients with long-term ventilator dependency (either by face mask or by tracheostomy tube).

### Nosocomial infections

Table 3 compares the results between the two groups.

**Table 3:** Nosocomial infections according to CDC criteria

	All patients (*n* = 378)	Transfused (*n* = 168)	Non-transfused (*n* = 210)	*P* value^*d*^
No. of patients with nosocomial infections	158 (41.8)	91 (54.2)	67 (31.9)	< 0.001^*a*^
No. of patients with MDR-NI	24 (15.2)	16 (17.6)	8 (11.9)	n.s.^*a*^
No. of nosocomial infections	210	133	77	–
No. of MDR-NI	28 (13.3)	20 (15.0)	8 (10.4)	n.s.^*a*^
No. of NI per patient	0.56 (± 0.78)	0.79 (± 0.92)	0.37 (± 0.57)	< 0.001^*c*^
No. of nosocomial infections by site/type				
Lower respiratory tract infection	72 (19.0)	48 (28.7)	24 (11.4)	< 0.001^*a*^
Ventilator-associated pneumonia	24 (6.4)	22 (13.1)	2 (1.0)	< 0.001^*a*^
Tracheobronchitis	48 (12.7)	26 (15.5)	22 (10.5)	n.s.^*a*^
Urinary tract infection	61 (16.1)	32 (19.0)	29 (13.8)	n.s.^*a*^
Gastroenteritis	32 (8.5)	20 (11.9)	12 (5.7)	n.s.^*a*^
Clostridium-associated infection	29 (7.7)	17 (10.1)	12 (5.7)	
Norovirus infection	3 (0.8)	3 (1.8)	0 (0.0)	
Decubitus infection	7 (1.9)	5 (3.0)	2 (1.0)	n.s.^*b*^
Catheter-related BSI	22 (5.8)	13 (7.7)	9 (4.3)	n.s.^*a*^
Other	16 (4.2)	15 (8.9)	1 (0.5)	< 0.001^*b*^
Biliary tract infection	1	1	0	
Lung abscess and empyema	1	1	0	
Endocarditis	1	1	0	
Laboratory-confirmed BSI	3	3	0	
Mediastinitis	1	1	0	
Peritonitis (primary/secondary)	3	3	0	
Skin and soft tissue infection	1	1	0	
Surgical site infection	5	4	1	
No. of isolated pathogens	241	153	88	–
No. of isolated MDR pathogens	30 (12.4)	21 (13.7)	9 (10.2)	n.s.^*a*^
Time to 1_st_ nosocomial infection (days)	14.7 (± 11.4)	16.4 (± 12.3)	12.5 (± 9.7)	0.030^*c*^

Legend

Results are presented as mean values (± standard deviation), number, or number (%)

*a*: Chi square test

*b*: Fisher’s exact test

*c*: Student’s *t*-test

*d: P* value for differences between transfused and non-transfused patients

*Abbreviations: No.* Number, *n.s.* not significant (*p* > 0.05), *BSI* Bloodstream infection, *MDR* Multidrug resistant, *NI* Nosocomial infection

One hundred fifty-eight patients, with a total of 210 nosocomial infections, were recorded. There were significantly more nosocomial infections in transfused patients. In particular, ventilator-associated pneumonia (VAP) was more common in the T group than in the NT group (13.1 and 1.0%, respectively; *p* <  0.001), with no significant differences detected in the other specific types of infections. There was only one patient with multiple occurrence of the same type of infection (two gastroenteritis infections).

Additional file 2: Tables S1 and S2 compare the number and percentage of different isolated pathogens between the two groups according to the type of nosocomial infection; Additional file 2: Figure S1 shows the percentage of different pathogens in patients with nosocomial infections [see Additional file 2].

### Results of multivariate analyses

Table 4 shows the results of the univariate and multivariate analyses of the impact of RBC transfusion on the selected endpoints.

**Table 4:** Impact of red blood cell transfusion on outcome parameters: Results of univariate and multivariate analyses

Outcome	Unadjusted OR (95% CI)	*P* value	Adjusted OR (95% CI)	*P* value
Weaning failure (Category 3c)	1.689 (1.085–2.629)	0.020	–	n.s.^*a*^
Hospital mortality	3.168 (1.625–6.177)	0.001	2.050 (0.995–4.224)	0.052^*a*^
Nosocomial infections	2.522 (1.625–6.177)	< 0.001	–	n.s.^*a*^
Outcome	Unadjusted β (95% CI)	*P* value	Adjusted β (95% CI)	*P* value
Weaning duration	12.386 (9.335–15.436)	< 0.001	12.386 (9.335–15.436)	< 0.001^*b*^
Hospital length of stay	18.664 (11.464–25.864)	< 0.001	16.116 (8.925–23.306)	< 0.001^*b*^

Legend

*a*: Logistic regression analysis

*b*: Linear regression analysis

Results were adjusted for baseline demographics and clinical characteristics (age, gender, body mass index, APACHE-II, Albumin, hemoglobin on admission, smoking history), comorbidities (Charlson comorbidity index, COPD, renal insufficiency), causes of acute respiratory failure (acute exacerbation of COPD, cardiopulmonary resuscitation, cardiac failure, surgery) as well as for mean hemoglobin, FFP/platelets, gastrointestinal bleeding, and other bleeding events in the course of weaning. The results for nosocomial infections were adjusted for weaning duration.

*Abbreviations: OR* Odds ratio, *95% CI* 95% confidence interval, *n.s*. not significant (*p* > 0.05)

RBC administration was independently correlated with the duration of weaning and hospital length of stay, but not with weaning failure (Category 3c); there was also a trend toward increased hospital mortality. Pearson correlation revealed a significant association of the number of transfused RBCs with the number of nosocomial infections (Pearson correlation coefficient 0.352; *p* < 0.001), but there was no independent correlation of RBCs with nosocomial infections in multivariate analysis.

Mean hemoglobin concentration in the course of weaning was correlated with weaning failure (Category 3c) in univariate, but not in multivariate analysis. There was also no independent correlation with the duration of weaning (adjusted β − 0.100, 95% CI -0.163 – 0.166; *p* = 0.683) or with hospital mortality (adjusted β 0.975, 95% CI 0.936–1.015; *p* = 0.420).

Hemoglobin concentration on the day of admission was an independent risk factor for hospital mortality (adjusted OR 0.956, 95% CI 0.924–0.989; *p* = 0.010). Analysis of the associated ROC shows a statistically significant result (AUC = 0.670, 95% CI 0.593–0.747; *p* < 0.001). Hemoglobin on admission of less than 8.5 g/dl was associated with increased mortality, but with low sensitivity of 67% and specificity of 63%. Based on the descriptive statistics, hospital mortality for hemoglobin on admission below 8.5 g/dl was 17.7%, whereas it was 8.5% for values above 8.5 g/dl.

APACHE-II score on admission was independently correlated with hospital length of stay (adjusted β 0.582, 95% CI 0.042–1.122; *p* = 0.035), and the CCI was an independent risk factor for hospital mortality (adjusted OR 1.249, 95% CI 1.094–1.425; *p* = 0.001).

Finally, the duration of weaning could be identified as the only independent risk factor for nosocomial infections (adjusted OR 1.065, 95% CI 1.045–1.076; *p* < 0.001).

## Discussion

The present study describes for the first time transfusion practice in prolonged mechanically ventilated, tracheotomized patients, treated at a specialized weaning center after surviving acute respiratory insufficiency. So far, there are only a few studies [3, 4] on this group of patients and the impact of RBC transfusion on weaning outcome, occurrence of nosocomial infections and survival has not yet been investigated.

An essential finding of the present study is that transfused patients differed significantly from those who were not transfused in the course of weaning regarding their clinical characteristics on admission. Patients in the T group appeared to be more diseased, recognizable by higher APACHE-II scores, lower levels of albumin and hemoglobin, and a larger proportion of cardiac comorbidities and renal insufficiency. The proportion of postoperative and in particular cardiac surgery patients was increased, in which postoperative blood loss may have led to lower hemoglobin levels on admission and more RBC transfusions in the course of weaning. These points may help explain the lower hemoglobin level on admission to the weaning center and the lower average hemoglobin concentration in transfused patients.

Anemia is common in the ICU [5], so the observed high prevalence on admission to the weaning center is not surprising. Only 18.8% of transfused patients showed hemoglobin concentrations below 7.0 g/dl prior to RBC administration and the mean pre-transfusion hemoglobin value was 7.5 g/dl (± 0.5 g/dl) which is, at first sight, higher then would be expected from recommendations of current guidelines [18, 21, 22]. This could be due to several reasons. First, the proportion of patients with concomitant coronary artery disease was 35.1%, and a total of 16.1% of patients in the T group had undergone cardiac surgery immediately prior to admission to the weaning center. In fact, current guidelines recommend a transfusion trigger of 8.0 g/dl in this subgroup of patients [18, 22]. Second, the lack of clinical studies regarding RBC administration in prolonged mechanically ventilated patients may have led to transfusions outside of evidence based guidelines. Current guidelines recommend a restrictive transfusion strategy in critically ill patients without concomitant cardiovascular diseases [21, 22], but it is still uncertain whether this can be applied simply to prolonged mechanically ventilated patients. Third, there were 17 patients in the T group with acute bleeding complications. In such patients, decreased hemoglobin values are not always evident and clinical parameters other than hemoglobin level, such as hemodynamic instability or shock, may have led to RBC transfusion [23]. Fourth, there has been a protocolized approach to RBC transfusion in all patients, but since this was a retrospective study, we cannot completely rule out deviations from the protocol [24]. Overall, this may explain the proportion of approximately 68% of patients transfused with a pre-transfusion hemoglobin level of 7–8 g/dl in the present study.

Transfused patients showed worse outcomes compared to non-transfused patients. Again, differences in clinical characteristics may have played a crucial role. Previous work has shown that a higher APACHE-II score in critically ill patients is associated with a longer ICU stay, prolonged mechanical ventilation, and increased mortality [25]. The significantly increased number of ventilator days on admission in the T group may be indicative of a more complicated course in the ICU. The fact that CCI was independently correlated with hospital mortality may indicate the importance of concomitant diseases in terms of survival. However, our results are in line with previous observational studies [12–15, 26, 27], where RBC administration after adjustment for differences in clinical characteristics between transfused and non-transfused patients, remained independently correlated with worse outcomes. In controlled trials with a well-balanced distribution of patient characteristics in the intervention and control group, comparing a restrictive and a liberal transfusion strategy in different ICU patients, contradictory results were found, which at present do not allow a definitive statement on the impact of RBC transfusion on the above mentioned outcomes [28–35].

Our results are not indicative of an independent association of RBC transfusion and weaning outcome. Although from a pathophysiological point of view improvement in oxygen transport capacity could facilitate sustained spontaneous breathing, there is currently no evidence, except for a small case series [7], to support the assumption that transfusions in anemic patients may reduce the duration of mechanical ventilation or affect weaning outcome [6].

Patients in the T group showed significantly more nosocomial infections; in particular VAP was more frequent. Previous studies that compared transfused with non-transfused patients in the ICU came to similar conclusions, identifying transfusions as an independent risk factor for nosocomial infections [12–14, 16, 26, 36, 37]. In the present study, there was no independent correlation of RBC transfusion and the occurrence of infectious complications. This may have different reasons. First, patients in both groups were transfused in the ICU so that a sustained immunomodulatory effect in the course of weaning cannot be ruled out. Second, all RBC transfusions were recorded during the weaning period regardless of the time of onset of nosocomial infection. Thus, some RBC units could have been administered after the occurrence of a nosocomial infection. Third, the only independent risk factor for nosocomial infections was the duration of weaning. A patient with a longer hospital stay is more likely to have a NI than a patient with a short stay, in particular the risk of VAP increases with prolonged ventilation [36]. Since the duration of weaning was significantly longer in the T group, this may help explain the higher percentage of NI and especially of VAP. In keeping with this hypothesis, there was no independent correlation of RBC transfusion with nosocomial infection after adjustment for weaning duration. Fourth, the way in which nosocomial infections were detected may have played a role. Previous work has used different definitions of nosocomial infections [12–14, 38], a significant proportion has been detected on the basis of clinical criteria, but without isolation of a pathogen. This increases the number of clinical events falsely detected as infection. In the present study, nosocomial infections were recorded based on the revised 2008 CDC criteria [20], validated for surveillance purposes [15, 39]. Based on these criteria, a proof of a pathogen is mandatory for the majority of the diagnoses. Again, this is more likely to underestimate the incidence of NI, because in the ICU in approximately 30% no pathogen detection succeeds [40]. Consequently, the frequencies of nosocomial infections vary between studies [13, 14, 39] and this may well have led to confounding of results within the studies. Looking at the randomized controlled trials that examined the impact of RBC transfusion on nosocomial infection rates, the picture is relatively consistent [30, 33, 41]. So far, no controlled study has proven a strong causal link between transfusions and nosocomial infections. Thus, all these trials probably indicate that surveillance studies may overestimate the risk of RBC administration for nosocomial infections.

Our study has several limitations. Due to the single-center design, the results are probably not transferable to other centers. Our results do not support a causal relationship between RBC administration and the significantly correlated endpoints; this requires a controlled trial. No information was provided on storage time, although all transfused RBCs were stored with saline-adenine-glucose-mannitol (SAG-M) and were leucocyte-depleted. We didn’t check medical records for anticoagulant/ antiplatelet therapy and apart from bleeding complications, other non-infectious complications in the course of weaning, which also could have had an impact on the endpoints we considered, were not recorded.

## Conclusions

Ninety-eight percent of prolonged mechanically ventilated patients showed anemia on admission to the weaning center. In multivariate analyses, RBC transfusion was independently correlated with weaning duration and hospital length of stay. There was also a trend toward increased hospital mortality, but there was no independent correlation with weaning outcome or nosocomial infections. Since transfused and non-transfused patients differed significantly regarding their clinical characteristics, RBC transfusion might be an indicator of disease severity rather than directly impacting patient prognosis.

## Supplementary information

**Additional file 1.** Supplemental methods; details on data collection and criteria for nosocomial infections.

**Additional file 2: Table S1**, Comparison of number and percentage of different isolated pathogens according to the type of nosocomial infection; **Table S2.** Comparison of number of different isolated MDR pathogens according to the type of nosocomial infection; **Figure S1.** Percentage of different pathogens in patients with nosocomial infections.

## Data Availability

The datasets used and/or analyzed during the current study are available from the corresponding author on reasonable request.

## Abbreviations

95% CI: 95% confidence interval
APACHE-II: Acute Physiology and Chronic Health Evaluation (score) 2
ARDS: Acute respiratory distress syndrome
AUC: Area under the curve
BSI: Bloodstream infection
CCI: Charlson comorbidity index
CDC: Centers for disease control and prevention
COPD: Chronic obstructive pulmonary disease
FFP: Fresh frozen plasma
ICU: Intensive care unit
I-HMV: Invasive home (mechanical) ventilation
MDR: Multidrug resistant
NI: Nosocomial infection
NIV: Non-invasive ventilation
NT group: Non-transfused group
OR: Odds ratio
RBC: Packed red blood cells
ROC: Receiver operating characteristic curve
SBT: Spontaneous breathing trial
SD: Standard deviation
T group: Transfused group
VAP: Ventilator-associated pneumonia
