# Supplementary material for: Anemia and red blood cell transfusion practice in prolonged mechanically ventilated patients admitted to a specialized weaning center: an observational study
Source: BMC Pulm Med. 2019 Dec 18;19:250. doi: 10.1186/s12890-019-1009-1 (PMC6921402; doi:10.1186/s12890-019-1009-1)
Supplement: Supplementary file 1 — Additional file 1. Supplemental methods; details on data collection and criteria for nosocomial infections. [file 12890_2019_1009_MOESM1_ESM.pdf]

Anemia and red blood cell transfusion practice in prolonged mechanically ventilated patients admitted to a specialized weaning center: an observational study.

Alessandro Ghiani, MD; Alexandros Sainis, MD; Georgios Sainis, MSc; and Claus Neurohr, MD.

Additional file 1

Supplemental methods (Page 2)

1. Details on data collection (Page 2)
2. Definitions of weaning outcome measures (Page 2)
3. Criteria for nosocomial infections (CDC) (Page 3)
4. Abbreviation list (Page 11)
5. References (Page 12)

Supplemental methods

1. Details on data collection

**Body mass index**	Body weight and height on admission were prospectively assessed and the body mass index (BMI) was calculated using the kg/m^2 formula.
**APACHE-II**	Acute physiology and health care evaluation score 2 was assessed based on patients medical records [1].
**Charlson comorbidity index**	Charlson comorbidity index (CCI) was assessed based on patients medical records [2].
**Causes of acute respiratory failure**	Causes of acute respiratory failure were assessed based on patients` medical records. If a combination of causes was present, only the one cause that was considered to be the main responsible was specified.
**Laboratory values**	Laboratory values were assessed based on patients` medical records.
**Smoking history**	Smoking history was assessed based on patients` medical records.
**COPD**	Chronic obstructive pulmonary disease (COPD) was assessed based on patients` medical records.
**Hepatopathy**	Hepatopathy (Cirrhosis, chronic viral hepatitis, secondary sclerosing cholangitis) was assessed based on patients` medical records.
**Renal insufficiency**	Renal function on admission was assessed using glomerular filtration rate (GFR), calculated by the Modification of Diet in Renal Disease (MDRD) formula [3]. In each case, the median of all GFR values was recorded on days 0–7 and days 8–15, respectively. The worse of the two values was used to estimate renal function based on a classification according to the Kidney Disease: Improving Global Outcomes (KDIGO) guideline [4].<br><br>This was done because there are frequently large fluid shifts in the first two weeks upon admission, as a result of a negative fluid balance in patients which are overhydrated when transferred from the intensive care unit to the weaning center.<br><br>GFR values calculated from creatinine trends that met the criteria of acute renal failure according to Acute Kidney Injury Network (AKIN) criteria [5] during the first 15 days after admission were excluded.
**Cardiac disease**	Patients` medical records were screened for documented coronary artery disease (diagnosis exclusively based on previous left-heart catheterization) and systolic left ventricular dysfunction (based on a recent echocardiographic examination not more than six months before).
**Diabetes mellitus**	DM was assessed based on patients` medical records.
**Neuromuscular disease**	Patients` medical records were screened for documented Parkinson`s disease, multiple sclerosis, myasthenia gravis, myotonic dystrophy, amyotrophic lateral sclerosis, and other types of neuromuscular disease.
**Interstitial lung disease**	Patients` medical records were screened for documented organizing pneumonia, hypersensitivity pneumonitis (HP), connective tissue disease-associated interstitial lung disease (CDT-ILD), sarcoidosis and idiopathic interstitial pneumonias such as idiopathic pulmonary fibrosis (IPF) or non-specific interstitial pneumonia (NSIP).
**Malignancy**	Patients` medical records were screened for documented active malignant disease present at the time of treatment.
**Immunosuppression**	Patients` medical records were screened for documented therapy with glucocorticoids (equivalent to prednisolone ≥ 20 mg per day for more than two weeks) during the course of weaning, chemotherapy or therapy with immunosuppressants not more than three months before, organ transplant, human immunodeficiency virus (HIV) infection category c/stage 3, splenectomy, and active hematologic malignancies.

2. Definitions of weaning outcome measures

**Weaning failure**	Failure was defined as Category 3c according to the German guideline on prolonged weaning [6], either transition to invasive home ventilation or death on ventilation during the treatment period.
**Weaning duration**	Time from admission to the weaning center to the point at which weaning was completed.<br><br>For Category 3a, equal to the time of the last mechanical ventilation episode,

	followed by permanent spontaneous breathing up to discharge from the weaning unit.<br><br>For Category 3b, equal to the time to transition to non-invasive home ventilation. This is not always the same as the time to extubation/decannulation. If decannulation has been delayed for other medical reasons, such as repeated bronchoscopic interventions for resection of subglottic tracheal stenosis prior to decannulation, then the time was chosen as the end of the weaning process, from which, due to the ventilatory capacity, a switch to NIV was considered.<br><br>For Category 3c, equal to the time to transition to invasive home ventilation (this was at the discretion of the treating physician) or death on ventilation during the treatment period.
**Hospital lenght of stay**	Time between admission to the weaning center and discharge from the hospital.
**Hospital mortality**	Proportion of patients deceased during their hospital stay.

3. Criteria for nosocomial infections (CDC) [7]

**Hospital Acquired Pneumonia**<br>*With common bacterial or filamentous fungal pathogens and specific lab findings*	
VAP	Pneumonia in patients who had a device to assist or control respiration continuously through a tracheostomy or by endotracheal intubation within the 48-hour period before the onset of infection, inclusive of the weaning period
	Patient with/without underlying diseases has 2/1 or more serial x-rays with one of the following:<br>- New or progressive and persistent infiltrate<br>- Consolidation<br>- Cavitation<br>- Pneumatoceles, in ≤ 1 y.o.<br><br>**AND**<br><br>At least one of the following:<br>- Fever (> 38°C/100.4°F) with no other cause<br>- Leukopenia (< 4.000 WBC/mm^3) or leukocytosis (> 12.000 WBC/mm^3)<br>- Altered mental status with no other cause, in ≥ 70. y.o.<br><br>**AND**<br><br>At least one of the following:<br>- New onset of purulent sputum, or change in character of sputum or ↑ respiratory secretions, or ↑ suctioning requirements<br>- New onset of worsening cough, or dyspnea, or tachypnea<br>- Rales or bronchial breath sounds<br>- Worsening gas exchange (e.g., O_2 desats [e.g., $PaO_2/FiO_2 \leq 240$], ↑ O_2 req, or ↑ ventilation demand)<br><br>**AND**<br><br>At least one of the following:<br>- Positive blood culture not related to another infection<br>- Positive pleural fluid culture<br>- Positive (semi)quantitative culture from minimally contaminated lower respiratory tract specimen (e.g., BAL or protected specimen brushing)<br>- ≥ 5% BAL-obtained cells contain intracellular bacteria on direct microscopic exam<br>- Histopathologic exam shows **one** of the following<br>o Abscess formation or foci of consolidation with intense PMN accumulation in bronchioles and alveoli<br>o Positive quantitative culture of lung parenchyma<br>o Evidence of lung parenchyma invasion by fungal hyphae or

	pseudohyphae
**Hospital Acquired Pneumonia**<br>*With viral, Legionella, Chlamydia, Mycoplasma, and other uncommon pathogens and specific lab findings*	
VAP	Pneumonia in patients who had a device to assist or control respiration continuously through a tracheostomy or by endotracheal intubation within the 48-hour period before the onset of infection, inclusive of the weaning period
	Patient with/without underlying diseases has <u>2/1 or more serial x-rays</u> with one of the following:<br>- New or progressive and persistent infiltrate<br>- Consolidation<br>- Cavitation<br>- Pneumatoceles, in ≤ 1 y.o.<br><br>**AND**<br><br>At least <u>one</u> of the following:<br>- Fever (> 38°C/100.4°F) with no other cause<br>- Leukopenia (< 4.000 WBC/mm^3) or leukocytosis (> 12.000 WBC/mm^3)<br>- Altered mental status with no other cause, in ≥ 70. y.o.<br><br>**AND**<br><br>At least <u>one</u> of the following:<br>- New onset of purulent sputum, or change in character of sputum or ↑ respiratory secretions, or ↑ suctioning requirements<br>- New onset of worsening cough, or dyspnea, or tachypnea<br>- Rales or bronchial breath sounds<br>- Worsening gas exchange (e.g., O_2 desats [e.g., PaO_2/FiO_2 ≤ 240], ↑ O_2 req, or ↑ ventilation demand)<br><br>**AND**<br><br>At least <u>one</u> of the following:<br>- Positive culture of virus or *Chlamydia* from respiratory secretions<br>- Positive detection of viral antigen or antibody from respiratory secretion (e.g., EIA, FAMA, shell vial assay, PCR)<br>- 4-fold rise in paired sera (IgG) for pathogen (e.g., *influenza viruses*, *Chlamydia*)<br>- Positive PCR for *Chlamydia* or *Mycoplasma*<br>- Positive micro-IF test for *Chlamydia*<br>- Positive culture or micro-IF of *Legionella* spp from respiratory secretions or tissue<br>- Detection of *Legionella pneumophila* serogroup 1 antigens in urine by RIA or EIA<br>- 4-fold rise in *L. pneumophila* antibody titer to > 1:128 in paired acute and convalescent sera by indirect IFA
**Hospital Acquired Pneumonia**<br>*Immunocompromised patients*	
VAP	Pneumonia in patients who had a device to assist or control respiration continuously through a tracheostomy or by endotracheal intubation within the 48-hour period before the onset of infection, inclusive of the weaning period
	Patient with/without underlying diseases has <u>2/1 or more serial x-rays</u> with one of the following:<br>- New or progressive and persistent infiltrate<br>- Consolidation<br>- Cavitation<br>- Pneumatoceles, in ≤ 1 y.o.<br><br>**AND**<br><br>At least <u>one</u> of the following:<br>- Fever (> 38°C/100.4°F) with no other cause

	- Altered mental status with no other cause, in ≥ 70. y.o.<br>- New onset of purulent sputum, or change in character of sputum or ↑ respiratory secretions, or ↑ suctioning requirements<br>- New onset of worsening cough, or dyspnea, or tachypnea<br>- Rales or bronchial breath sounds<br>- Worsening gas exchange (e.g., O_2 desats [e.g., $PaO_2/FiO_2 \leq 240$], ↑ O_2 req, or ↑ ventilation demand)<br>- Hemoptysis<br>- Pleuritic chest pain<br><br>**AND**<br><br>At least <u>one</u> of the following:<br>- Matching positive blood and sputum cultures with *Candida* spp<br>- Evidence of fungi or *Pneumocystis carinii* from minimally contaminated lower respiratory tract specimen (e.g., BAL or protected specimen brushing) from <u>one</u> of the following:<br>o Direct microscopic exam<br>o Positive culture of fungi
**Hospital Acquired Pneumonia**<br>*Clinically defined pneumonia*	
VAP	Pneumonia in patients who had a device to assist or control respiration continuously through a tracheostomy or by endotracheal intubation within the 48-hour period before the onset of infection, inclusive of the weaning period
	Patient with/without underlying diseases has <u>2/1 or more serial x-rays</u> with one of the following:<br>- New or progressive and persistent infiltrate<br>- Consolidation<br>- Cavitation<br>- Pneumatoceles, in ≤ 1 y.o.<br><br>**AND**<br><br>At least <u>one</u> of the following:<br>- Fever (> 38°C/100.4°F) with no other cause<br>- Leukopenia (< 4.000 WBC/mm^3) or leukocytosis (> 12.000 WBC/mm^3)<br>- Altered mental status with no other cause, in ≥ 70. y.o.<br><br>**AND**<br><br>At least <u>two</u> of the following:<br>- New onset of purulent sputum, or change in character of sputum or ↑ respiratory secretions, or ↑ suctioning requirements<br>- New onset of worsening cough, or dyspnea, or tachypnea<br>- Rales or bronchial breath sounds<br>- Worsening gas exchange (e.g., O_2 desats [e.g., $PaO_2/FiO_2 \leq 240$], ↑ O_2 req, or ↑ ventilation demand)
**Lower respiratory tract infection, other than pneumonia**<br>*Bronchitis, tracheobronchitis, and tracheitis*	
	Patient without clinical or radiological evidence of pneumonia<br><br>**AND**<br><br><u>Two</u> of the following:<br>- Fever (> 38°C/100.4°F) with no other cause<br>- New onset of worsening cough<br>- New onset of purulent sputum, or ↑ respiratory secretions<br>- Rales or bronchial breath sounds<br><br>**AND**

	One of the following:<br>- Positive (semi)quantitative culture from minimally contaminated lower respiratory tract specimen (e.g., tracheal secretion or BAL)<br>- Positive detection of antigen from respiratory secretion
**Lung abscess and empyema**	
	At least one of the following signs or symptoms with no other recognized cause:<br>- Fever (> 38°C)<br>- Cough, sputum production<br><br>**AND**<br><br>High suspicion for abscess on radiographic examination<br><br>**AND**<br><br>All of the following:<br>- Drainage of pus from suspected lung abscess or empyema by puncture or surgical operation<br>- Confirmed etiologic agent visible in gram staining or pathogen isolated from pus culture
**Symptomatic urinary tract infection**<br>*Catheterized patients*	
	At least one of the following with no other recognized cause:<br>- Fever (> 38°C)<br>- Suprapubic tenderness<br>- Urgency, frequency, or dysuria<br><br>**AND**<br><br>Urine culture with $\geq 10^5$ colonies/mL of no more than two species of microorganisms
	At least one of the following with no other recognized cause:<br>- Fever (> 38°C)<br>- Suprapubic tenderness<br>- Urgency, frequency, or dysuria<br><br>**AND**<br><br>Urine culture with $\geq 10^3$ but $< 10^5$ colonies/mL of no more than two species of microorganisms<br><br>**AND**<br><br>At least one of the following:<br>- Positive dipstick for leukocyte esterase and/or nitrate<br>- Pyuria (urine specimen with ≥ 10 WBC/mm^3 or ≥ 3 WBC/high-power field of unspun urine)<br>- Organisms seen on Gram`s stain of unspun urine
**Symptomatic urinary tract infection**<br>*Noncatheterized patients (> 48 hours)*	
	At least one of the following with no other recognized cause:<br>- Fever (> 38°C) in < 65. y.o.<br>- Suprapubic tenderness<br>- Urgency, frequency, or dysuria<br><br>**AND**

	Urine culture with ≥ 10^5 colonies/mL of no more than two species of microorganisms
	At least one of the following with no other recognized cause:<br>- Fever (> 38°C) in < 65. y.o.<br>- Suprapubic tenderness<br>- Urgency, frequency, or dysuria<br><br>**AND**<br><br>Urine culture with ≥ 10^3 but < 10^5 colonies/mL of no more than two species of microorganisms<br><br>**AND**<br><br>At least one of the following:<br>- Positive dipstick for leukocyte esterase and/or nitrate<br>- Pyuria (urine specimen with ≥ 10 WBC/mm^3 or ≥ 3 WBC/high-power field of unspun urine)<br>- Organisms seen on Gram`s stain of unspun urine
**Other infections of the urinary tract**<br>*Kidney, ureter, bladder, urethra, or tissue surrounding the retroperitoneal or perinephric space*	
	At least one of the following:<br>1) Patient has organisms isolated from culture of fluid (other than urine) or tissue from affected site.<br>2) Patient has an abscess or other evidence of infection seen on direct examination, during a surgical operation, or during a histopathologic examination<br>3) Patient has at least two of the following signs or symptoms with no other recognized cause: fever (> 38°C), localized pain, or localized tenderness at involved site **AND** at least one of the following:<br>o Purulent drainage from affected site<br>o Organisms cultured from blood that are compatible with suspected site of infection<br>o Radiographic evidence of infection (e.g., abnormal ultrasound, CT-scan, MRI)
**Bloodstream infection (LC-BSI)**<br>*Laboratory-confirmed BSI (primary sepsis)*	
Clinical	Laboratory-confirmed bloodstream infection in a patient without an evident focus
	1) Patient has a pathogen cultured from one or more blood cultures<br>2) Patient has at least one of the following symptoms (fever > 38°C, shivering, hypotonia) **AND** has a recognized pathogen (defined as a microorganism not usually regarded as a common skin contaminant, i.e., diphtheroids, Bacillus species, Propionibacterium species, coagulase-negative staphylococci, or micrococci) cultured from at least 2 blood cultures drawn on separate occasions
**Bloodstream infection (CR-BSI)**<br>*Catheter-related BSI*	
Clinical	Bloodstream infection in a patient with one or more intravascular-access devices for more than 72 hours
	Clinical signs of infection with at least one of the following criteria:<br>- Fever (> 38°C) with no other cause<br>- Chills<br>- Hypotension (systolic pressure <100 mmHg) or need for vasopressors<br><br>**AND**<br><br>Positive blood culture of peripheral blood (venapuncture) or blood obtained from other catheter line<br><br>**AND**

	At least <u>one</u> of the following:<br>- Positive catheter tip culture for same pathogen that was recovered from blood culture (species and antibiogram)<br>- Purulent drainage from affected catheter line
**Sinusitis**	
	Patient with clinical suspicion for sinusitis with at least one of the following signs or symptoms with no other recognized cause:<br>- Fever (> 38°C)<br>- Leukocytosis (> 12.000 WBC/mm^3)<br><br>**AND**<br><br>At least <u>one</u> of the following criteria:<br>- Positive transillumination with air-fluid level<br>- radiologically suspected for sinusitis (CT, ultrasound)<br><br>**AND**<br><br>Positive culture (> 1000 colonies/ml) of purulent discharge from sinus cavity plus > 5 PMN per oil immersion field
**Mediastinitis**	
	Mediastinitis must meet at least 1 of the following criteria:<br>1. Patient has organisms cultured from mediastinal tissue or fluid obtained during a surgical operation or needle aspiration.<br>2. Patient has evidence of mediastinitis seen during a surgical operation or histopathologic examination.<br>3. Patient has at least 1 of the following signs or symptoms with no other recognized cause: fever (.388C), chest pain, or sternal instability **AND** at least <u>one</u> of the following:<br>o Purulent discharge from mediastinal area<br>o Organisms cultured from blood or discharge from mediastinal area<br>o Mediastinal widening on x-ray
**Decubitus infection**	
	Patient has at least <u>two</u> of the following signs or symptoms with no other recognized cause:<br>- Redness<br>- Tenderness<br>- Swelling of decubitus wound edges<br><br>**AND**<br><br>At least <u>one</u> of the following:<br>- Organisms cultured from properly collected fluid or tissue (see comments)<br>- Organisms cultured from blood
Comments	- Purulent drainage alone is not sufficient evidence of an infection<br>- Organisms cultured from the surface of a decubitus ulcer are not sufficient evidence that the ulcer is infected. A properly collected specimen from a decubitus ulcer involves needle aspiration of fluid or biopsy of tissue from the ulcer margin
**Primary peritonitis**<br>*Spontaneous bacterial peritonitis*	
Clinical setting	Patients presenting with an infection of the peritoneal fluid in the absence of a gastrointestinal perforation, abscess, or other localized infection within the gastrointestinal tract

	At least <u>two</u> of the following signs or symptoms with no other recognized cause:<br>- Fever (> 38°C)<br>- Abdominal pain in more than 1 quadrant (not localized)<br>- Ileus<br>- Feeding intolerance<br>- Inflammatory peritoneal fluid (> 500 leukocytes/ml with neutrophil predominance)<br>- Presence of a positive Gram stain in peritoneal fluid<br><br>**AND**<br><br>Isolation of microbial pathogens (in peritoneal fluid or blood)
**Secondary peritonitis**	
Clinical setting	Patients presenting with an infection of the peritoneal space following perforation, abscess formation, ischemic necrosis, or penetrating injury of the intra-abdominal contents
	At least <u>two</u> of the following signs or symptoms with no other recognized cause:<br>- Fever (> 38°C)<br>- Abdominal pain in more than 1 quadrant (not localized)<br>- Ileus<br>- Feeding intolerance<br><br>**AND**<br><br>Isolation of one or more microbial pathogens found in the peritoneum or the blood 24 hrs after a gastrointestinal perforation of the stomach, esophagus or duodenum, or any perforation of the small bowel distal to the ligament of Treitz
**Endocarditis**	
Clinical setting	Patients presenting with SIRS/sepsis without an evident clinical focus, or with persistent SIRS/sepsis despite adequate therapy for any suspected alternative source
	Endocarditis of a natural or prosthetic heart valve must meet at least <u>one</u> of the following criteria:<br>- Patient has organisms cultured from valve or vegetation<br>- Patient has 2 or more of the following signs or symptoms with no other recognized cause: fever (> 38°C), new or changing murmur, embolic phenomena, skin manifestations (i.e., petechiae, splinter hemorrhages, painful subcutaneous nodules), congestive heart failure, or cardiac conduction abnormality<br><br>**AND**<br><br>At least <u>one</u> of the following:<br>- Organisms cultured from 2 or more blood cultures<br>- Organisms seen on Gram's stain of valve when culture is negative or not done<br>- Valvular vegetation seen during a surgical operation or autopsy<br>- Positive antigen test on blood or urine (eg, H influenzae, S pneumoniae, N meningitidis, or Group B Streptococcus)<br>- Evidence of new vegetation seen on echocardiogram<br><br>**AND**<br><br>If diagnosis is made ante mortem, physician institutes appropriate antimicrobial therapy
**Gastroenteritis**	
	Gastroenteritis must meet at least <u>one</u> of the following criteria:<br><br>1) Patient has an acute onset of diarrhea (liquid stools for more than 12

	hours) with or without vomiting or fever (> 38°C) and no likely noninfectious cause (e.g., diagnostic tests, therapeutic regimen other than antimicrobial agents, acute exacerbation of a chronic condition, or psychologic stress)<br><br>2) Patient has at least <u>two</u> of the following signs or symptoms with no other recognized cause: nausea, vomiting, abdominal pain, fever (> 38°C), or headache and at least 1 of the following:<br>o An enteric pathogen is cultured from stool or rectal swab<br>o An enteric pathogen is detected by routine or electron microscopy<br>o An enteric pathogen is detected by antigen or antibody assay on blood or feces<br>o Evidence of an enteric pathogen is detected by cytopathic changes in tissue culture (toxin assay)<br>o Diagnostic single antibody titer (IgM) or 4fold increase in paired sera (IgG) for pathogen
**Surgical site infections**<br>*Superficial wounds*	
Clinical setting	Patients presenting with symptoms or signs of wound infection within 30 days following surgery or trauma
	Infection involves only skin and subcutaneous tissue of the incision<br><br>**AND**<br><br>Patient has at least <u>one</u> of the following:<br>- Purulent drainage from the superficial incision<br>- Organisms isolated from an aseptically obtained culture of fluid or tissue from the superficial incision<br>- At least 1 of the following signs or symptoms of infection: pain or tenderness, localized swelling, redness, or heat, and superficial incision is deliberately opened by surgeon and is culture positive or not cultured. A culture-negative finding does not meet this criterion.<br>- Diagnosis of superficial incisional SSI by the surgeon or attending physician
**Surgical site infections**<br>*Deep wounds*	
Clinical setting	Infection occurs within 30 days after the operative procedure if no implant is left in place or within 1 year if implant is in place and the infection appears to be related to the operative procedure
	Infection involves deep soft tissues (e.g., fascial and muscle layers) of the incision<br><br>**AND**<br><br>Patient has at least <u>one</u> of the following:<br>- Purulent drainage from the deep incision but not from the organ/space component of the surgical site<br>- A deep incision spontaneously dehisces or is deliberately opened by a surgeon and is culture-positive or not cultured when the patient has at least 1 of the following signs or symptoms: fever (> 38°C), or localized pain or tenderness. A culture-negative finding does not meet this criterion.<br>- An abscess or other evidence of infection involving the deep incision is found on direct examination, during reoperation, or by histopathologic or radiologic examination<br>- Diagnosis of a deep incisional SSI by a surgeon or attending physician
**Osteomyelitis**	
	At least <u>one</u> of the following criteria:<br>- Patient has organisms cultured from bone<br>- Patient has evidence of osteomyelitis on direct examination of the bone

	during a surgical operation or histopathologic examination<br>- Patient has at least 2 of the following signs or symptoms with no other recognized cause: fever (> 38°C), localized swelling, tenderness, heat, or drainage at suspected site of bone infection<br><br>**And**<br><br>At least one of the following:<br>- Organisms cultured from blood<br>- Positive blood antigen test (e.g., H influenzae, S pneumoniae)<br>- Radiographic evidence of infection (e.g., abnormal findings on x-ray, CT scan, MRI, radiolabel scan [gallium, technetium, etc.])

Abbreviation list

APACHE II: Acute Physiology and Chronic Health Evaluation (score) 2

AKIN: Acute Kidney Injury Network

BMI: Body mass index

CCI: Charlson Comorbidity Index

CDC: Centers for disease control and prevention

CDT-ILD: Connective tissue disease-associated interstitial lung diseases

COPD: Chronic obstructive pulmonary disease

GFR: Glomerular filtration rate

HIV: Human immunodeficiency virus

HP: Hypersensitivity pneumonitis

IPF: Idiopathic pulmonary fibrosis

KDIGO: Kidney Disease – Improving Global Outcomes

MDRD: Modification of Diet in Renal Disease

NSIP: Non-specific interstitial pneumonia

VAP: Ventilator-associated pneumonia

References

1. Knaus WA, Draper EA, Wagner DP, et al. APACHE-II: A severity disease classification system. Crit Care Med 1985;13:818-29.
2. Charlson ME, Pompei P, Ales KL, et al. A new method of classifying prognostic comorbidity in longitudinal studies: development and validation. J Chronic Dis 1987;40:373-83.
3. Levey AS, Bosch JP, Lewis JB, et al. A More Accurate Method To Estimate Glomerular Filtration Rate from Serum Creatinine: A New Prediction Equation. Ann Intern Med 1999;130:461-70.
4. KDIGO 2012 Clinical Practice Guideline for the Evaluation and Management of Chronic Kidney Disease. Kidney Int Suppl 2013;3:1-163.
5. KDIGO clinical practice guideline for acute kidney injury. Kidney Int Suppl 2012;2:1-138.
6. Schönhofer B, Geiseler J, Dellweg D, et al. Prolonged Weaning: S2k-Guideline Published by the German Respiratory Society. Pneumologie 2014;68:19-75.
7. Horan TC, Andrus M, Dudeck MA. CDC/NHSN surveillance definition of health care–associated infection and criteria for specific types of infections in the acute care setting. Am J Infect Control 2008;36:309-32.
