# Supplementary material for: Anemia and red blood cell transfusion practice in prolonged mechanically ventilated patients admitted to a specialized weaning center: an observational study
Source: BMC Pulm Med. 2019 Dec 18;19:250. doi: 10.1186/s12890-019-1009-1 (PMC6921402; doi:10.1186/s12890-019-1009-1)
Supplement: Supplementary file 2 — Additional file 2: Table S1, Comparison of number and percentage of different isolated pathogens according to the type of nosocomial infection; Table S2. Comparison of number of different isolated MDR pathogens according to the type of nosocomial infection; Figure S1. Percentage of different pathogens in patients with nosocomial infections. [file 12890_2019_1009_MOESM2_ESM.pdf]

Anemia and red blood cell transfusion practice in prolonged mechanically ventilated patients admitted to a specialized weaning center: an observational study.

Alessandro Ghiani, MD; Alexandros Sainis, MD; Georgios Sainis, MSc; and Claus Neurohr, MD.

Additional file 2

1. **Table S1 (Page 2)**
2. **Table S2 (Page 3)**
3. **Figure S1 (Page 3)**

1. Table S1: Comparison of number and percentage of different isolated pathogens according to the type of nosocomial infection

Pathogen	Group	VAP	TB	UTI	Gastroenteritis	Decubitus infection	CR-BSI	Other
P. aeruginosa	**T**	6 (30.0)	12 (36.4)	6 (16.7)	-	2 (40.0)	-	3 (12.5)
	**NT**	-	7 (28.0)	11 (31.4)	-	-	-	-
E. faecium	**T**	-	-	1 (2.8)	-	2 (40.0)	2 (13.3)	5 (20.8)
	**NT**	-	-	4 (11.4)	-	-	-	1 (100.0)
E. faecalis	**T**	-	-	2 (5.6)	-	-	2 (13.3)	2 (8.3)
	**NT**	-	-	3 (8.6)	-	1 (25.0)	2 (22.2)	-
S. epidermidis	**T**	-	-	-	-	-	8 (53.3)	1 (4.2)
	**NT**	-	-	-	-	-	1 (11.1)	-
S. aureus	**T**	4 (20.0)	6 (18.2)	1 (2.8)	-	-	1 (6.7)	2 (8.3)
	**NT**	1 (50.0)	6 (24.0)	1 (2.9)	-	-	3 (33.3)	-
E. coli	**T**	-	6 (18.2)	8 (22.2)	-	-	-	2 (8.3)
	**NT**	-	3 (12.0)	7 (20.0)	-	-	-	-
Enterobacter spp.	**T**	1 (5.0)	-	2 (5.6)	-	1 (20.0)	-	3 (12.5)
	**NT**	1 (50.0)	2 (8.0)	1 (2.9)	-	1 (25.0)	-	-
K. pneumoniae	**T**	1 (5.0)	4 (12.1)	3 (8.3)	-	-	-	1 (4.2)
	**NT**	-	3 (12.0)	4 (11.4)	-	-	-	-
K. oxytoca	**T**	1 (5.0)	-	1 (2.8)	-	-	-	-
	**NT**	-	-	1 (2.9)	-	1 (25.0)	1 (11.1)	-
S. marcescens	**T**	2 (10.0)	-	4 (11.1)	-	-	-	-
	**NT**	-	1 (4.0)	-	-	-	-	-
C. freundii	**T**	-	-	1 (2.8)	-	-	-	-
	**NT**	-	-	-	-	-	-	-
P. mirabilis	**T**	-	-	1 (2.8)	-	-	-	-
	**NT**	-	2 (8.0)	3 (8.6)	-	1 (25.0)	-	-
A. baumanii	**T**	2 (10.0)	1 (3.0)	-	-	-	-	-
	**NT**	-	1 (4.0)	-	-	-	-	-
S. maltophilia	**T**	2 (10.0)	4 (12.1)	-	-	-	-	-
	**NT**	-	-	-	-	-	-	-
Candida spp.	**T**	-	-	3 (8.3)	-	-	1 (6.7)	-
	**NT**	-	-	-	-	-	1 (11.1)	-
C. difficile	**T**	-	-	-	17 (85.0)	-	-	-
	**NT**	-	-	-	12 (100.0)	-	-	-
Norovirus	**T**	-	-	-	3 (15.0)	-	-	-
	**NT**	-	-	-	-	-	-	-
Others	**T**	1 (5.0)	-	3 (8.3)	-	-	1 (6.7)	5 (20.8)
	**NT**	-	-	-	-	-	1 (11.1)	-
No pathogen detected	**T**	8	-	2	-	-	-	3
	**NT**	-	1	1	-	-	-	-
**Summary**	**T**	**20 (100)**	**33 (100)**	**36 (100)**	**20 (100)**	**5 (100)**	**15 (100)**	**24 (100)**
	**NT**	**2 (100)**	**25 (100)**	**35 (100)**	**12 (100)**	**4 (100)**	**9 (100)**	**1 (100)**

Legend

Results are presented as number or number (%).

Abbreviations: T, transfused group; NT, non transfused group; VAP, Ventilator-associated pneumonia; TB, Tracheobronchitis; UTI, Urinary tract infection; CR-BSI, Catheter-related blood stream infection; P. aeruginosa, Pseudomonas aeruginosa; E. faecium, Enterococcus faecium; E. faecalis, Enterococcus faecalis; S. epidermidis, Staphylococcus epidermidis; S. aureus, Staphylococcus aureus; E. coli, Escherichia coli; spp., species; K. pneumoniae, Klebsiella pneumoniae; K. oxytoca, Klebsiella oxytoca; S. marcescens, Serratia marcescens, C. freundii, Citrobacter freundii; P. mirabilis, Proteus mirabilis; A. baumanii, Acinetobacter baumanii; S. maltophilia, Stenotrophomonas maltophilia; C. difficile, Clostridium difficile; NI, Nosocomial infection.

2. Table S2: Comparison of number of different isolated MDR pathogens according to the type of nosocomial infection

Pathogen	Group	Drug Resistance	VAP	TB	UTI	Decubitus infection	CR-BSI	Other	Summary
P. aeruginosa	**T**	3MRGN	2	1					**3**
		4MRGN	1		1	1		1	**4**
	**NT**	3MRGN		1	1				**2**
		4MRGN			2				**2**
S. epidermidis	**T**	MRSE					6		**6**
	**NT**	MRSE					1		**1**
S. aureus	**T**	MRSA	1	1					**2**
	**NT**	MRSA					1		**1**
E. coli	**T**	3MRGN						1	**1**
		ESBL							
	**NT**	3MRGN							
		ESBL			1				**1**
Enterobacter spp.	**T**	Amp-C		1	2	1		1	**5**
	**NT**	Amp-C			1	1			**2**
**Summary**	**T**		**4**	**3**	**3**	**2**	**6**	**3**	**21**
	**NT**		**0**	**1**	**5**	**1**	**2**	**0**	**9**

Legend
Results are presented as number.

Abbreviations: T, transfused group; NT, non transfused group; MDR, Multidrug resistant; VAP, Ventilator-associated pneumonia; TB, Tracheobronchitis; UTI, Urinary tract infection; CR-BSI, Catheter-related blood stream infection; 3MRGN, multiresistant gram negative bacteria non-susceptible to three specific groups of antibiotics; 4MRGN, multiresistant gram negative bacteria non-susceptible to four specific groups of antibiotics; Amp-C, ampC β-lactamases producing antimicrobial resistant bacteria; MRSA, methicillin-resistant staphylococcus aureus; ESBL, extended spectrum β-lactamases producing antimicrobial resistant bacteria; MRSE, methicillin-resistant staphylococcus epidermidis.

3. Figure S1: Percentage of different pathogens in patients with nosocomial infections

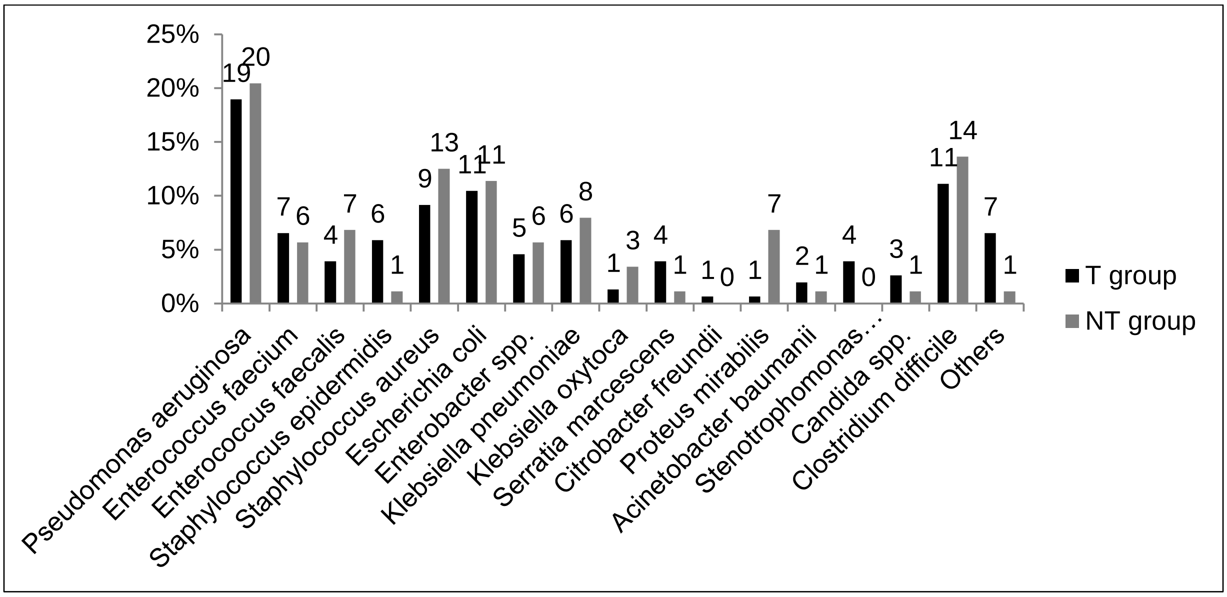

Legend
Abbreviations: spp., species; T group, transfused group; NT group, non-transfused group; Staphylococcus …, Staphylococcus epidermidis; Stenotrophomonas …, Stenotrophomonas maltophilia.
